# Cooperation of arbuscular mycorrhizal fungi and bacteria to facilitate the host plant growth dependent on soil pH

**DOI:** 10.3389/fmicb.2023.1116943

**Published:** 2023-02-20

**Authors:** Zengwei Feng, Xiaodi Liu, Yongqiang Qin, Guangda Feng, Yang Zhou, Honghui Zhu, Qing Yao

**Affiliations:** ^1^Key Laboratory of Agricultural Microbiomics and Precision Application (MARA), Guangdong Provincial Key Laboratory of Microbial Culture Collection and Application, Key Laboratory of Agricultural Microbiome (MARA), State Key Laboratory of Applied Microbiology Southern China, Institute of Microbiology, Guangdong Academy of Sciences, Guangzhou, China; ^2^College of Horticulture, Guangdong Province Key Laboratory of Microbial Signals and Disease Control, Guangdong Engineering Research Center for Litchi, South China Agricultural University, Guangzhou, China

**Keywords:** arbuscular mycorrhizal fungi, cooperation, *Gigaspora*, plant growth promotion, *Sphingomonas*, adaptation to native soils

## Abstract

Almost all plants grow well in their native soils. We hypothesized that soil microbes promote the growth of their hosts in native soils by the example of soil pH. Here, bahiagrass (*Paspalum notatum* Flugge) indigenous to subtropical soils was grown in the native soil (the original pH = 4.85) or in pH-adjusted soils with sulfur (pH = 3.14 or 3.34) or calcium hydroxide (pH = 6.85, 8.34, 8.52 or 8.59). Plant growth, soil chemical property, and microbial community composition were characterized to reveal the microbial taxa promoting plant growth in the native soil. Results showed that shoot biomass was the highest in the native soil, while both the decrease and increase in the soil pH reduced the biomass. Compared with other soil chemical properties, soil pH was the top edaphic factor contributing to the differentiation in arbuscular mycorrhizal (AM) fungal and bacterial communities. The top 3 most abundant AM fungal OTUs belonged to *Glomus*, *Claroideoglomus*, and *Gigaspora*, while the top 3 most abundant bacterial OTUs belonged to *Clostridiales*, *Sphingomonas*, and *Acidothermus*, respectively. Regression analyses between microbial abundances and shoot biomass revealed that the most abundant *Gigaspora* sp. and *Sphingomonas* sp. were the most promotive fungal and bacterial OTUs, respectively. The application of these two isolates to bahiagrass solely or in combination indicated that *Gigaspora* sp. was more promotive than *Sphingomonas* sp. across the soil pH gradient, and they positively interacted to enhance biomass only in the native soil. We demonstrate that microbes cooperate to facilitate host plants to grow well in their native soils with the original pH. Meanwhile, a high-throughput sequencing-guided pipeline to efficiently screen for beneficial microbes is established.

## Introduction

The plant rhizosphere is a hotspot for microbes, harboring a diverse array of fungi, bacteria, protists, nematodes, and other soil microorganisms (Pathan et al., 2020). Many rhizosphere microbes can confer benefits to the host plants under a variety of stresses conditions (e.g., unsuitable soil pH) and thus show a promotive effect on the host plant growth in response to these stresses (Luo et al., 2019; Pandey and Gupta, 2019; Feng et al., 2020a,^2022^). For instance, arbuscular mycorrhizal (AM) fungi are important and symbiotic fungi in the rhizosphere, establishing symbiosis with most terrestrial plant species and conferring benefits to host plants in most studies (Smith and Read, 2010). In addition, plant growth-promoting rhizobacteria (PGPRs) have been largely proven to promote plant growth with varied mechanisms, e.g., production of indole-3-acetic acid (IAA), siderphores, antagonism to pathogens, and 1-aminocyclopropane-1-carboxylate (ACC) deaminase (Subiramani et al., 2020; Feng et al., 2022). Therefore, the application of these beneficial microbes to promote the host plant growth in native soils is a priority due to their adaptation to the native soil conditions, especially the original soil pH.

Most studies focus on how microbes promote plant fitness in response to stress conditions. However, plant associated microbes also play an active role in promoting host plant growth under the native soils whose properties have not been substantially altered (Johnson et al., 2010; Mishra and Sundari, 2013; Chialva et al., 2018; Camarena-Pozos et al., 2019). In other words, these associated microbes can facilitate plants to grow well in native soils. For example, microbiomes in the native soils of tomatoes could elicit an immune system protecting tomato plants from bacterial wilt disease, while no protection was observed in disinfected soils; however, the inoculation of AM fungus restored resistance to the disease (Chialva et al., 2018). In another experiment with plants (agaves and cacti) endemic to drylands, the rhizosphere bacterial isolates improved the growth and development of hosts *via* the emission of microbial volatile organic compounds (Camarena-Pozos et al., 2019). These PGPRs represent the desirable microbial resources for agricultural production. In general, this evidence indicates that plants can recruit appropriate microbial assembly to facilitate their growth not only in stress conditions (e.g., unsuitable soil pH) but also in native soils (e.g., suitable soil pH).

Soil pH is one of the major edaphic factors affecting plant growth as well as the community compositions and functions of their rhizosphere microbes. In general, both plants and their rhizosphere microbes have the optimal soil pH condition, and the unsuitable soil pH (higher or lower soil pH) may cause their growth and function to be inhibited. For instance, low pH can significantly decrease root growth, root cell viability, nutrient uptake by reducing net proton release, and inhibit the beneficial functions (e.g., nutrient acquisition) of the root-associated microbes (Yan et al., 1992; Wang et al., 2017; Feng et al., 2022). It is estimated that acidic soil is present throughout the tropical and subtropical regions of southern China, representing 21.8% of the total national area (Liu et al., 2020). Therefore, the unsuitable soil pH, especially low soil pH, is a non-negligible soil problem in agricultural production in southern China.

Since rhizosphere microbes may promote host plant growth in native soils, uncovering the beneficial microbial taxa can be of great significance. However, little information is available thus far on how to accurately identify and isolate these beneficial taxa with high efficiency. The routine strategy is to isolate the culturable taxa from targeted environments as much as possible and then to evaluate the functionality of each isolate. Although a large number of beneficial isolates have been screened and evaluated on plates, many are low-effective or ineffective after being released into native soils (Collavino et al., 2010; Manzoor et al., 2017; Valetti et al., 2018). Consequently, a highly efficient and focused strategy is needed to guide the identification and isolation of beneficial microbial taxa.

In most cases, plants grow best in their native soils, which is probably the result of environmental selection and evolutionary adaptation (Hoffmann and Willi, 2008; Anderson et al., 2011). However, whether and how soil microbes promote the growth of their hosts in native soils by the example of soil pH are largely unknown. We hypothesized that the rhizosphere microbiome of host plants could facilitate them to adapt to the original soil pH and that high-throughput sequencing can be employed to guide the identification and isolation of these beneficial microbial taxa (e.g., AM fungi and PGPRs) contributing to the facilitation. In this study, we used a subtropical soil as the original soil (pH = 4.85) and established seven soils with different pH levels (3.14∼8.59) by adding sulfur or calcium hydroxide. We grew bahiagrass (*Paspalum notatum* Flugge) in these soils, which is native to the original soil, and further investigated the fluctuations of microbial community across the soil pH gradient based on high-throughput sequencing. In the second experiment, isolated microbial taxa were inoculated into sterilized soils with a pH gradient, and the plant growth promotion was estimated. By combining the high-throughput sequencing and microbial culture method, we aimed to identify the AM fungal and bacterial taxa facilitating the adaptation of the plant to its native soil pH.

## Materials and methods

### Pot soil preparation

The original soil was collected from a subtropical orchard (23°9’30.3” N, 113°21’37.2” E), air-dried, and sieved through a 2 mm pore-sized mesh. Soil chemical properties were as follows: soil pH, 4.85; soil organic matter (SOM), 15.70 g/kg; total nitrogen (TN), 0.81 g/kg; total phosphorus (TP), 0.22 g/kg; total potassium (TK), 4.56 g/kg; available nitrogen (AN), 76.80 mg/kg; available phosphorus (AP), 12.50 mg/kg; available potassium (AK), 54.10 mg/kg; exchangeable calcium (ECa), 1.58 mg/kg; available sulphur (AS), 10.90 mg/kg; and exchangeable aluminum (EAl), 115.75 mg/kg.

Soil pH was set as the only edaphic variable in this study. The soil pH was adjusted according to Soti et al. (2015). In detail, soils were mixed thoroughly with sulfur powder at rates of 1.0 or 2.0 g/kg or with calcium hydroxide powder at rates of 1.5, 3.0, 4.5, or 7.5 g/kg, respectively. The adjusted soils were irrigated and incubated in a greenhouse. The soil moisture content was kept at 18% by the weighting method every 2 days. After 2 months of incubation, the soil pH values were determined to be 3.14, 3.34, 6.85, 8.34, 8.52, and 8.59, respectively, with an original soil pH of 4.85. To ensure sufficient nutrients for plant growth, all soils were supplemented with an additional 200 mg/kg N [CO(NH_2_)_2_], 50 mg/kg P (KH_2_PO_4_), and 150 mg/kg K (K_2_SO_4_) for the pot experiments.

### Experimental design, plant growth, and harvest

Plastic pots were used as containers with 320 g of soil per pot. Seven treatments corresponding to seven pH levels were established. There were three biological replicates for each treatment. Bahiagrass (*Paspalum notatum* Flugge), native to subtropical soils, was selected as the test plant, which is widely grown as a cover crop in orchards (Cui et al., 2015; Prince et al., 2018). Previous work demonstrated the preference of bahiagrass for the soil with a low pH (Prince et al., 2018). Plant seeds were gained from Cui et al. (2015) and Zhou et al. (2019), surface-sterilized and pregerminated. Six germinating seeds were sown in each pot and thinned to three seedlings after seedling emergence. All 21 pots were placed in a greenhouse with natural light at 22∼30°C and 60∼80% relative humidity. Pots were irrigated to a soil moisture content of 18% with deionized water by weighing every 2 days.

Plants were harvested after 4 months of growth. Shoots were gently excised and cleaned with tap water. Shoot dry weight was measured after oven drying at 65°C. Roots were collected from the soil, cleaned, and divided into two aliquots. One aliquot was stored at 4°C for mycorrhizal colonization measurement. The other aliquot was quick-frozen in liquid nitrogen immediately and stored at −80°C for root DNA extraction.

The soil in each pot was homogenized and divided into three aliquots. One aliquot was air-dried and used for analysis of soil chemical properties. The second aliquot was stored at 4°C for trap culturing of AM fungal spores and soil bacterial isolation. The last aliquot was quick-frozen in liquid nitrogen immediately and stored at −80°C for soil DNA extraction.

### Analysis of soil chemical properties

Soil pH was determined with a water/soil ratio of 2.5/1. SOM, TN, TP, TK, AN, AP, and AK were determined as described by Zhou et al. (2017). Briefly, SOM was determined by titration after wet oxidation with H_2_SO_4_ and K_2_Cr_2_O_7_ (Heanes, 1984). TN, TP, and TK were determined using the Kjeldahl method, the molybdenum blue colorimetric method, and the flame photometric method (Analytik Jena novAA^®^ 350, Germany), respectively. AN, AP, and AK were determined by the alkaline hydrolysis diffusion method, the molybdenum blue colorimetric method, and the flame photometric method, respectively. ECa, AS, and EAl were determined by the atomic absorption spectrometry method, barium sulfate turbidity method, and spectrophotometric method, respectively.

### Mycorrhizal colonization

Root segments were stained with trypan blue according to Phillips and Hayman (1970) and then mounted onto a slide and observed under an optical microscope (Olympus, BX53). Mycorrhizal colonization was quantified according to Trouvelot et al. (1986). Mycorrhizal colonization is classed into 0 to 5, including 0, 1, 5, 30, 70, and 95%; arbuscule abundance is classed into A0 to A3, including 10, 50, and 100%. The whole mycorrhizal colonization level (including arbuscule abundance) was assessed by microscopic examination and raw results of 30 root segments were used for calculations in the software. Mycorrhiza frequency (F%, the frequency of mycorrhiza in total root segments), mycorrhizal colonization intensity (M%, intensity of mycorrhizal colonization in total root segments), and arbuscular abundance (A%, intensity of mycorrhizal colonization in colonized root segments) were calculated with the computer program “Mycocalc.”^1^

### DNA extraction, PCR amplification of 18S and 16S rRNA genes and amplicon sequencing

DNA was extracted from 15 to 50 mg of roots or 400 mg of well-mixed soil using the DNeasy^®^ PowerPlant^®^ Pro Kit or PowerSoil^®^ DNA Isolation Kit (QIAGEN, Germany) according to the manufacturer’s protocols, respectively. To reduce soil heterogeneity again, soil DNA was extracted twice and pooled for later experiments. The concentration, quality, and purity of the extracted DNA were determined with an ultramicro spectrophotometer (ThermoFisher Scientific, NanoDrop2000) and 1% (w/v) agarose gel electrophoresis. DNA was stored at −80°C for further analysis.

Two primer pairs AML1/AML2 (Lee et al., 2008) and AMV4.5NF/AMDGR (Sato et al., 2005) were used to amplify the AM fungal 18S ribosomal RNA gene region with nested PCR. Van Geel et al. (2014) indicated that the complementary specificity of the two primer pairs made the primer combination highly suitable for tandem use in covering the diversity of AM fungal communities. First, PCR was performed with 12.5 μL of 2 × KAPA HiFi HotStart ReadyMix, 5 μL of each primer (1 μM), and 2.5 μL of DNA template (5 ng/μL) under the following thermal cycling conditions: initial denaturation at 95°C for 3 min, followed by 35 cycles of denaturation at 95°C for 45 s, annealing at 51°C for 40 s, and extension at 72°C for 1 min, and a final extension at 72°C for 5 min. The first amplification products were diluted with PCR grade water (1:10), and a 1 μL subsample was used as a template for the second PCR amplification under the same reaction as in the first PCR amplification. The second thermal cycling conditions were as follows: initial denaturation at 95°C for 3 min, followed by 35 cycles of denaturation at 95°C for 40 s, annealing at 59°C for 45 s, and extension at 72°C for 1 min, and a final extension at 72°C for 5 min. The second amplification products were separated by 1.5% (w/v) agarose gel electrophoresis, and the bands were excised, and purified with 0.8 × volume AMPure XP Beads. This primer pair was comprised of Illumina sequencing adapters, 8-bp-long barcodes, and transposase sequences for library preparation. The latter PCR was performed with 25 μL 2 × KAPA HiFi HotStart ReadyMix, 5 μL of each primer (10 μM), 5 μL DNA template and 10 μL of PCR grade water under the following thermal cycling conditions: initial denaturation at 95°C for 3 min, followed by 8 cycles of denaturation at 95°C for 30 s, annealing at 55°C for 30 s, and extension at 72°C for 30 s, and a final extension at 72°C for 10 min. The amplification products were purified with 1 × volume AMPure XP Beads and were evaluated using the Qubit dsDNA HS Assay Kit (Invitrogen, Carlsbad, CA, USA) and Qubit 2.0 Fluorometer (Applied Biosystems, Carlsbad, CA, USA). These products from same sample were mixed at equimolar concentrations as a sequencing pool. Ten nanograms of sequencing pool was selected and subjected to sequencing on an Illumina MiSeq PE300 platform using the MiSeq Reagent Kit version 3 (Illumina, San Diego, CA, USA). Sequencing was performed by the Chinese National Human Genome Center in Shanghai, China.

The V3–V4 hypervariable regions of bacterial 16S ribosomal RNA gene were amplified using the primer pair 338F and 806R with sequencing adapters and barcodes. The PCR was performed in a 25 μL mixture containing 12.5 μL of the PCR premix FastPfu DNA polymerase, 1 μL of each primer (10 μM), and 1 μL of DNA template (approximately 20 ng of DNA) under the following thermal cycling conditions: initial denaturation at 95°C for 3 min, followed by 24 cycles of denaturation at 94°C for 30 s, annealing at 57°C for 90 s, and extension at 72°C for 60 s, and a final extension at 72°C for 5 min. The PCR products were extracted from 1.5% agarose gel and purified with a GeneJET Gel Extraction Kit (Thermo Scientific). The sequencing library was generated with the MetaVxTM Library Preparation Kit (GENEWIZ, Inc., South Plainfield, USA). The quality and concentration of the library was determined by an Agilent 2100 Bioanalyzer (Agilent Technologies, Palo Alto, CA, USA) and Qubit 2.0 Fluorometer. These amplicons were sequenced on the Illumina MiSeq PE300 platform (GENEWIZ, Suzhou, China).

Raw paired-ended sequences were merged, and then sequences ≥ 200 bp with an average quality score > 20 and without ambiguous bases were acquired using the Quantitative Insights Into Microbial Ecology (QIIME) pipeline (v1.9.1) (Caporaso et al., 2010). Potential chimeric sequences were identified and removed using the UCHIME algorithm in the USEARCH program (Edgar et al., 2011). The remaining sequences were assigned to the same operational taxonomic units (OTUs) at a 97% similarity using the UCLUST method (Edgar, 2010). OTUs with fewer than ten sequences were removed to reduce the risk of artificially inflating the richness as a result of a sequencing error (Xiang et al., 2014). The taxonomy of each representative and high-quality sequence was annotated against the Silva (SSU132) 16S rRNA database for bacteria and the MaarjAM database for AM fungi, respectively (Öpik et al., 2010; Xu et al., 2018). The OTU community matrices were used for the downstream analysis.

### Isolation and identification of AM fungus and bacterium

According to the results of the high-throughput sequencing, we performed regression analysis between the plant biomass and fungal or bacterial OTU abundances. The OTUs, whose abundances ranked in the top 10 and showed a positive relationship with the plant biomass, were selected as the target. To identify the closest taxonomy, the representative sequence of each targeted OTU was identified by sequence alignment at the National Center for Biotechnology Information (NCBI). Finally, one AM fungal OTU (OTU4, the second highest abundance and the maximum regression coefficient) and one bacterial OTU (OTU3, the first highest abundance and the maximum regression coefficient) were picked out, taxonomically belonging to *Gipaspora* and *Sphingomonas*, respectively.

To isolate the target AM fungus, trap culturing was first conducted to enrich spores for 5 months (Liu and Wang, 2003), and AM fungal spores were then collected with the wet-sieving and decanting method (Gerdemann and Nicolson, 1963). AM fungi were identified according to the spore morphology^2^ in combination with a molecular technique. Briefly, a single spore was rinsed in sterile distilled water three times in a PCR tube and crushed with a pipette tip under sterile conditions. DNA was extracted with 30 μL of TE buffer solution containing 20% Chelex 100 (Sigma) at 100°C for 5 min. After centrifugation, the supernatant was stored at −20°C. Nest PCR was then performed with the two primer pairs AML1/AML2 and AMV4.5NF/AMDGR. These sequences were aligned with that of *Gigaspora* OTU4, and spores corresponding to the matching sequence were considered as the target AM fungal taxa for later culture.

*Sphingomonas* was isolated on R2A solid medium with the dilution plating method according to Vanbroekhoven et al. (2004). Yellow-pigmented colonies were picked out and cultured in R2A liquid medium. Bacterial cells were then collected, and DNA was extracted with 100 μL of TE buffer solution containing 20% Chelex 100 (Sigma) at 100°C for 5 min. RCR reaction was performed with the universal 16S primer pair 27f/1492r (Luo et al., 2019), which covers the full length of products amplified with the primer pair 338F and 806R employed in high-throughput sequencing. The PCR products were sequenced, and the resultant sequences were aligned with that of *Sphingomonas* OTU3. The isolate corresponding to the matching sequence was considered to be the target bacterial taxa for later proliferation.

### Pot experiment to verify the promotive effects of the target fungal and bacterial taxa

The target AM fungal taxa, namely *Gigaspora* OTU4, was proliferated from 10 spores with clover (*Trifolium repens* L.) as the host plant in 300 mL pots containing 250 g growth substrate. In detail, a mixture (v/v = 2:1) of river sand and diatomite (2∼4 mm) was autoclaved (121°C, 2 h, twice) and then used as growth substrate. To ensure the symbiotic relationship between clover root and AM fungus was established as soon as possible, clover seeds were sown and cultivated for 1 month. And then, ten spores (approximately 300 μm) of the same morphology were picked out and inoculated into the growth substrate near the roots. Pots were irrigated to a soil moisture content of 15% by weighing every 2 days and fertilized with Hoagland nutrient solution (5 mL 100 μM P) once a week. After 4 months of the inoculation, approximately 1,300 spores of *Gigaspora* OTU4 were produced per pot and used as fungal inoculum. Twenty spores were randomly picked out and verified by a molecular technique as mentioned above. The target bacterial taxa, namely *Sphingomonas* OTU3, was proliferated in R2A liquid culture as bacterial inoculum (Luo et al., 2019).

The soil pH was adjusted with sulfur powder or calcium hydroxide powder as described previously to verify the growth promotion effects of these two target microbes (*Gigaspora* OTU4 and *Sphingomonas* OTU3) on bahiagrass at different pH levels. Five soil pH levels were thus established, e.g., pH = 3.44, pH = 4.17, pH = 4.85 (original soil), pH = 6.37, and pH = 7.44. At each pH level, a completely randomized experimental design was set up, including four inoculation treatments (no inoculation, bacterial inoculation, fungal inoculation, and dual inoculation), with each treatment comprised of four biological replicates. All soils were sterilized with ^60^Co-γ radiation at 30 kGy. For fungal and dual inoculation, 50 spores were added into the middle layer of soil. For bacterial and dual inoculation, 10 mL of bacterial suspension was added into the soil after seedling emergence for 10 days, while 10 mL of sterile distilled water was added into the soil simultaneously for non-bacterial inoculation.

All 80 pots were placed in a plant growth chamber for 6 weeks, with a photoperiod of 16 h/8 h (day/night), temperature of 28°C/20°C (day/night) and light intensity of 180 μmol/m^2^/s. Pots were irrigated to a soil moisture content of 18% with deionized water by weighing every 2 days. Plants were harvested after 6 weeks of growth, and the biomass was recorded.

### Statistical analysis

All results are the average of three or four replicates ± standard error. Analysis of variance (ANOVA), Tukey’s honestly significant difference (HSD) test, and independent sample *t* test were conducted with SPSS statistical software (v21.0, SPSS Inc., Chicago, IL, USA) after data normal distribution test and homogeneity of variances test. The data that did not meet the analysis standards needed to be transformed before further analysis.

The following analyses and visualizations were mainly performed in R version 3.5.1 (R Core Team, 2013) unless otherwise stated. Microbial community analysis was performed with the package “vegan” (Oksanen et al., 2017). All samples were normalized to the lowest number (8,327 AM fungal sequences in roots, 8,811 AM fungal sequences in soils, and 36,912 bacterial sequences in soils) of the sequence in certain sample by random subsampling without replacement by using the function “rrarefy” (Sosa-Hernández et al., 2018). Alpha diversity was calculated, including observed OTUs, Chao1 index, Shannon-wiener index, and Simpson’s diversity index. To analyze the specific and shared species across seven treatments, an UpSet plot was performed with TBtools (Chen et al., 2020). Bray-Curtis dissimilarity matrix was generated with the functions “decostand” and “vegdist” (Bray and Curtis, 1957), non-metric multidimensional scaling (NMDS) was generated with the function “metaMDS,” and analysis of similarities (ANOSIM) was carried out with the function “anosim” and 999 permutations. Canonical correspondence analysis (CCA) was performed to determine the effects of soil chemical properties on AM fungal communities in the roots and soils as well as on the bacterial community in the soils with the functions “cca” and “envfit.”

To visualize the associations between the AM fungal OTUs and bacterial OTUs in the network interface, these correlation matrices were constructed by calculating Spearman’s rank correlation with their relative abundances. To reduce network complexity, these OTUs had an average relative abundance of greater than 0.5%. Then, only significant correlations (correlation coefficient ρ > 0.6 and *P*-value < 0.01) were used for further analysis of the correlation network (Barberán et al., 2012). The probability was adjusted using the Benjamini-Hochberg method (namely, function “fdr” in R) to reduce false-positive results. Finally, analyses of the correlation network were visualized using Gephi software (Bastian et al., 2009).

All the raw sequence data from this study have been deposited in the Sequence Read Archive (SRA) at the National Center for Biotechnology Information (NCBI) database under BioProject IDs PRJNA691052 and PRJNA691053.

## Results

### Plant growth and mycorrhizal colonization across the soil pH gradient

**FIGURE 1:**
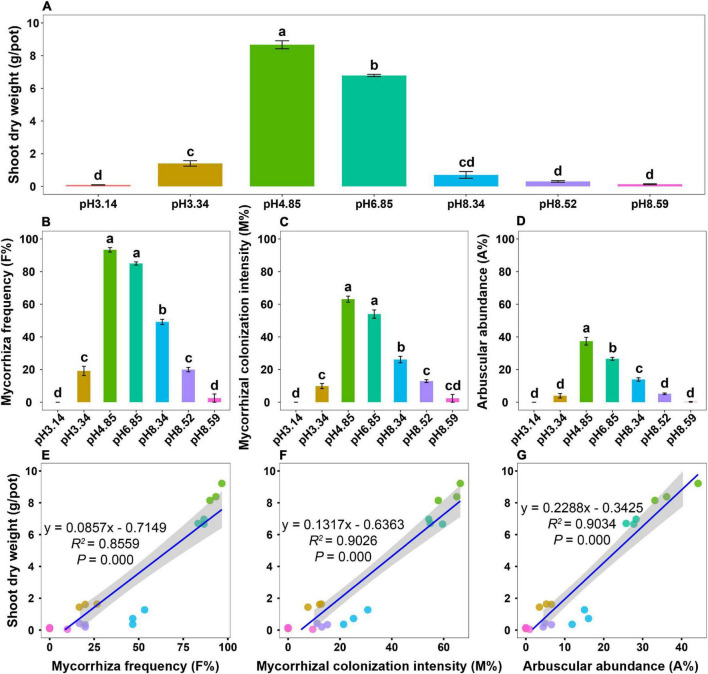
Plant growth and mycorrhizal colonization of bahiagrass across the soil pH gradient. **(A)** Shoot dry weight; **(B)** mycorrhizal frequency; **(C)** mycorrhizal colonization intensity; **(D)** arbuscular abundance; **(E)** regression analysis between the shoot dry weight and mycorrhizal frequency; **(F)** regression analysis between the shoot dry weight and mycorrhizal colonization intensity; **(G)** regression analysis between the shoot dry weight and arbuscular abundance. Bars (mean ± standard error, *n* = 3) with different letters are significantly different (*P* < 0.05) by Tukey’s HSD test.

After 4 months of growth, plant growth performance varied drastically across the soil pH gradient, and all roots were colonized by AM fungi (Supplementary Figure 1). The shoot biomass was the greatest in the native soil (pH = 4.85) but significantly decreased as the soil pH either decreased or increased (Figure 1A). Mycorrhizal colonization, including mycorrhizal frequency (F%), mycorrhizal colonization intensity (M%), and arbuscular abundance (A%), peaked at a pH of 4.85 (Figures 1B–D). The dynamic patterns of mycorrhizal colonization across the soil pH gradient were similar to those of the shoot biomass (Figures 1A–D). Regression analysis revealed that the shoot biomass significantly correlated with mycorrhizal colonization (Figures 1E–G), which highlights the involvement of AM fungi in promoting host plant growth.

### AM fungal and bacterial communities in response to the soil pH gradient

High-throughput sequencing indicated that eight AM fungal genera were detected, among which *Rhizophagus* was present in the soils but absent in the roots. The average relative abundances of *Glomus* across the soil pH gradient were the highest both in roots (43.40%) and soils (41.05%), followed by *Claroideoglomus*, *Scutellospora*, and *Gigaspora* (Supplementary Figure 2). Intriguingly, relative abundances of *Glomus* in roots or soils at a pH of 4.85 were the lowest compared to those at other pH levels, which possibly suggests a negative relationship between the *Glomus* abundance and plant growth across the present pH range. We performed regression analysis between the abundances of AM fungal genera and shoot biomass. The results indicated that there was no significant correlation between the abundances of all seven AM fungal genera in the roots and shoot biomass, but there was a significant positive relationship between the abundances of *Claroideoglomus* and *Gigaspora* in the soils and shoot biomass, and a significant negative relationship between the abundances of *Glomus* in the soils and shoot biomass (Supplementary Figure 3).

**FIGURE 2:**
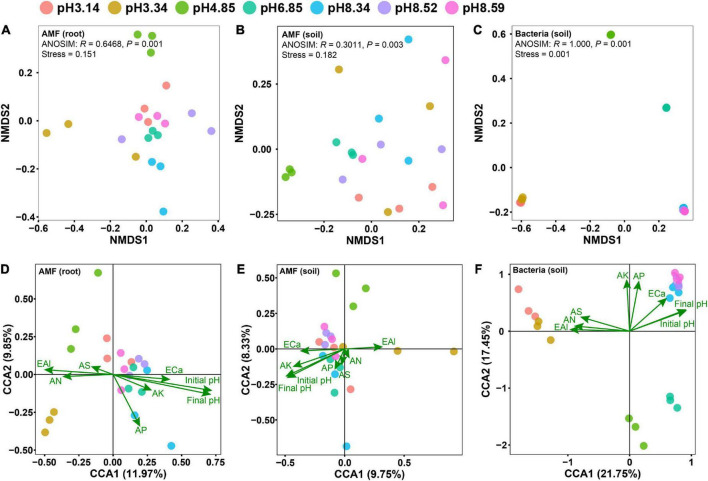
Differences in microbial community structures as revealed by nonmetric multidimensional scaling (NMDS) analysis **(A–C)** and the effects of edaphic factors on the microbial community as revealed by canonical correspondence analysis (CCA) **(D–F)**. Panels **(A,D)** arbuscular mycorrhizal (AM) fungal community in roots; panels **(B,E)** AM fungal community in soils; panels **(C,F)** bacterial community in soils. Initial pH, the soil pH before planting bahiagrass; Final pH, the soil pH after planting bahiagrass for 4 months; SOM, soil organic matter; TN, total nitrogen; TP, total phosphorus; TK, total potassium; AN, available nitrogen; AP, available phosphorus; AK, available potassium; ECa, exchangeable calcium; AS, available sulphur; EAl, exchangeable aluminum.

Non-metric multidimensional scaling (NMDS) analyses revealed that the effect of the soil pH on the bacterial community structure was greater than that on the AM fungal community structure (Figures 2A–C). Although the AM fungal community in the native soil separated from others, the AM fungal communities in the modified soils did not differ from each other (Figures 2A, B). It is notable that the soil pH was the sole edaphic factor significantly affecting the AM fungal communities in roots and soils, and it was the most important edaphic factor affecting the bacterial community in soils (Figures 2D–F and Supplementary Table 1).

High-throughput sequencing indicated that the AM fungal species richness (OTU number) and alpha diversity in the roots were the highest when plants were grown in the native soil (pH = 4.85), while these two parameters in the soil were the highest when plants were grown at a pH of 6.85, followed by a pH of 4.85 (Supplementary Table 2). However, the bacterial species richness and alpha diversity were peaked at a pH of 6.85, followed by that at a pH of 8.34 (Supplementary Table 2).

**FIGURE 3:**
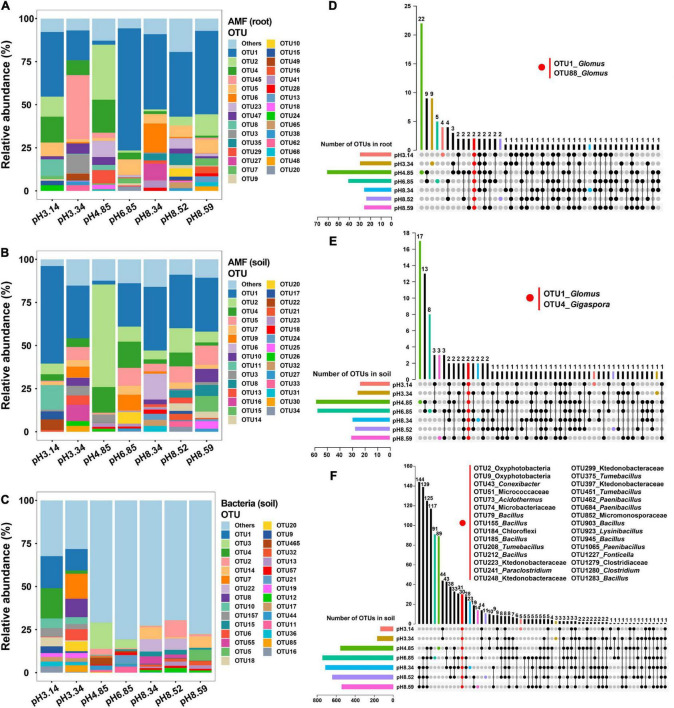
Relative abundances of arbuscular mycorrhizal (AM) fungal operational taxonomic units (OTUs) and bacterial OTUs as affected by the soil pH and the corresponding common OTUs as revealed by an UpSet plot. **(A,D)** AM fungal community in roots; **(B,E)** AM fungal community in soils; **(C,F)** bacterial community in soils.

We explored the microbial community structure at the OTU level across the soil pH gradient. The top 30 AM fungal OTU abundances represented 88.5∼90.2% of the total AM fungal OTU abundance, while the top 30 bacterial OTU abundances were only 38.4% of the total bacterial OTU abundance (Figure 3 and Supplementary Tables 3–5). OTU1, OTU2, and OTU4 were the three most abundant AM fungal OTUs in both roots and soils (Supplementary Tables 3, 4), while OTU1, OTU3 and OTU4 were the three most abundant bacterial OTUs in soils (Supplementary Table 5).

### The abundant AM fungal and bacterial taxa promoting plant growth

**FIGURE 4:**
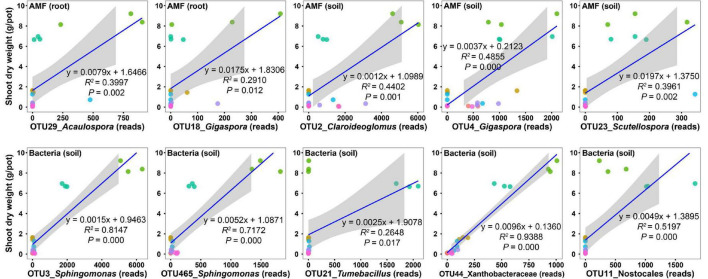
Regression analysis between the shoot dry weight and the abundances of microbial operational taxonomic units (OTUs). Only those OTUs that have top 30 abundances and show a significant and positive relationship with the shoot dry weight are presented.

To uncover the potential taxa promoting host plant growth, we performed regression analysis between the shoot biomass and the abundances of the top 30 OTUs (Supplementary Tables 3–5). For AM fungal OTUs in roots, the abundances of two OTUs (OTU29 and OTU18) were significantly and positively related to the shoot biomass (Figure 4 and Supplementary Table 3); however, their relative abundances were only 1.46 and 0.53%, respectively (Supplementary Table 3), indicating that they were rare species in the community and thus presumably are low effect in promoting plant growth. For AM fungal OTUs in soils, the abundances of three OTUs (OTU2, OTU4, and OTU23) were significantly and positively related to the shoot biomass (Figure 4 and Supplementary Table 4). Their relative abundances were 14.28, 7.33, and 0.71%, respectively (Supplementary Table 4). Although the relative abundance of OTU2 was two-fold that of OTU4, the regression coefficient of OTU4 (*y* = 0.0037x + 0.2123, *R*^2^ = 0.4855, *P* = 0.000) was three-fold that of OTU2 (*y* = 0.0012x + 1.0989, *R*^2^ = 0.4402, *P* = 0.001) (Figure 4 and Supplementary Table 4). Therefore, we speculated that OTU4 was more promotive for plant growth than OTU2, at least in the native soil. Sequence similarity indicated that AM fungal OTU4 was an isolate belonging to *Gigaspora*.

For bacterial OTUs in soils, the abundances of five OTUs (OTU3, OTU465, OTU21, OTU44, and OTU11) were significantly and positively related to the shoot biomass (Figure 4 and Supplementary Table 5), with relative abundances of 2.98, 0.79, 0.75, 0.69, and 0.67%, respectively (Supplementary Table 5). Sequence similarity indicated that bacterial OTU3 was an isolate belonging to *Sphingomonas*.

### Isolation of targeted AM fungal and bacterial taxa

We enriched AM fungal spores with trap culturing for 5 months, and 28 isolates were collected based on the spore morphological features. According to the sequence similarity, 5 isolates belonged to *Acaulospora*, 6 to *Claroideoglomus*, 7 to *Gigaspora*, 7 to *Scutellospora*, and 3 to *Glomus*. The sequence of one isolate belonging to *Gigaspora* showed the highest sequence similarity (99.54%) with that of OTU4, presumably designated as *Gigaspora* OTU4. *Gigaspora* OTU4 was propagated with pure spores for 4 months for the following inoculation experiment (Supplementary Figure 4).

Similarly, 56 isolates of *Sphingomonas* were collected with selective R2A medium. Sequencing results indicated that 98.6% (55 out of 56 isolates) of them belonged to *Sphingomonas*, and one isolate shared 99.82% sequence similarity with OTU3, presumably designated as *Sphingomonas* OTU3. *Sphingomonas* OTU3 was cultured for the following experiment (Supplementary Figure 4).

### Plant growth promotion by *Gigaspora* OTU4 and *Sphingomonas* OTU3

**FIGURE 5:**
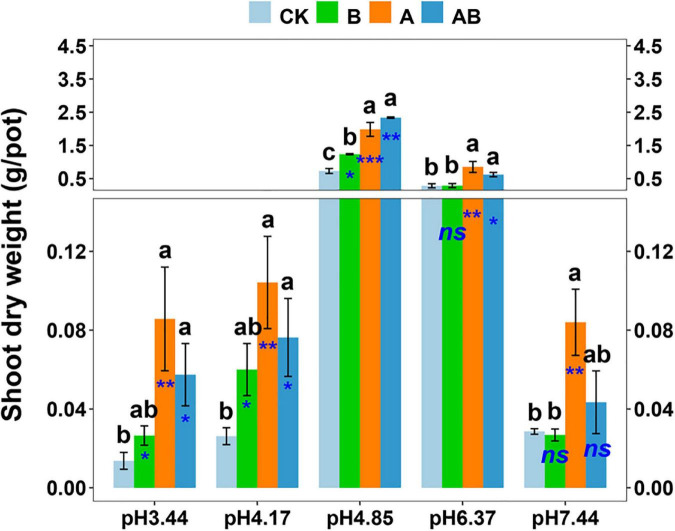
Shoot dry weight as affected by microbial inoculation across the soil pH gradient. CK, control without inoculation; B, inoculation with bacterial isolate *Sphingomonas* OTU3; A, inoculation with AM fungal isolate *Gigaspora* OTU4; AB, dual inoculation with both bacterial and AM fungal isolates. Bars (mean ± standard error, *n* = 4) with different letters are significantly different (*P* < 0.05) by Tukey’s HSD test. Independent sample *t* test is performed to evaluate the significance between CK treatment and the other three treatments. *P < 0.05; **P < 0.01; ***P < 0.001; *ns*, non-significance.

In the inoculation experiment, the plant growth promotion by *Gigaspora* OTU4 and/or *Sphingomonas* OTU3 was confirmed. The dynamics of the shoot biomass across the soil pH range were similar to those in the previous experiment, with the plant growth in the native soil (pH = 4.85) ranking first (Figure 5). *Gigaspora* OTU4 significantly increased the shoot biomass at five pH levels, with contribution rates of 63.1∼84.0%; *Sphingomonas* OTU3 significantly increased the shoot biomass at three pH levels (pH = 3.44, 4.17, 4.85), with contribution rates of 40.9∼56.3% (Figure 5).

In dual inoculation treatment, *Gigaspora* OTU4 and *Sphingomonas* OTU3 exhibited a positive interaction only in the native soil (pH = 4.85) (Table 1). The shoot biomass of dual inoculation treatment was higher than that of *Gigaspora* OTU4 treatment (increasing rate of 17.8%) and *Sphingomonas* OTU3 treatment (increasing rate of 88.9%) in the native soil (pH = 4.85). Furthermore, network analysis indicated a direct positive interaction between *Gigaspora* OTU4 and *Sphingomonas* OTU3 (Supplementary Figure 5), probably suggesting a direct interaction between them.

**TABLE 1:** Contribution rates (%) of *Gigaspora* OTU4 or *Sphingomonas* OTU3 to the shoot biomass across the soil pH gradient.

pH range	Inoculation			Fungal-bacterial interaction
	***Sphingomonas* OTU3**	***Gigaspora* OTU4**	**Dual**	
pH = 3.44	48.3	84.0	76.1	negative
pH = 4.17	56.3	74.9	65.7	negative
pH = 4.85	40.9	63.1	68.7	positive
pH = 6.37	1.9	66.4	53.9	negative
pH = 7.44	−6.7	66.0	34.1	negative

The contribution rate is calculated as 100% × (biomass of inoculated plants – biomass of non-inoculated plants)/biomass of inoculated plants. When the contribution of dual inoculation was lower than that of either bacterial or fungal inoculation, the interaction is defined as negative; otherwise, the interaction is defined as positive.

## Discussion

### Microbe-mediated plant adaptation to soil environments

The facilitation of host plants to resist adverse conditions (e.g., salinity, drought, and low fertility) by the associated microbes has been well-documented (Kang et al., 2019; Khoshru et al., 2020; Wissuwa et al., 2020; Zhang et al., 2020); however, the role of microbes in helping their host thrive in the native soil is less reported. Bahiagrass is a subtropical grass native to acidic soil. In this study, bahiagrass achieved the best growth performance in the native soil (the original pH 4.85) despite the presence of soil microbes, indicating that the pH of 4.85 rather than a lower or higher pH is optimal for this plant species. Therefore, the native soil with a pH of 4.85 provided the optimal edaphic conditions for bahiagrass, while other pH levels represented stress conditions in this study. Some plant species develop unique mechanisms to adapt to the native soil, such as biomass allocation between below- vs. above-ground nitrogen status (Avolio and Smith, 2013; Lu et al., 2019). In this study, we demonstrated that both AM fungus (*Gigaspora* OTU4) and bacterium (*Sphingomonas* OTU3) facilitated bahiagrass to adapt to the native soil because both of them increased the shoot biomass at a pH of 4.85. Furthermore, *Gigaspora* OTU4 and *Sphingomonas* OTU3 positively interacted to promote the shoot biomass only in the native soil with the original soil pH. Our study indicated for the first time that soil microbes can be an important contributor to their host fitness in the native soil other than under stress conditions.

Our results revealed that some AM fungal isolates (e.g., *Gigaspora* OTU4) and bacterial isolates (e.g., *Sphingomonas* OTU3) helped their host thrive in the native soil. AM fungi are ubiquitous symbiotic fungi in association with the roots of most terrestrial plants and promote the host growth (Smith and Read, 2010). Enhanced nutrient (especially phosphorus) uptake of roots by AM fungi is the key mechanism by which AM fungi promote the growth of host plants (Deguchi et al., 2012). Our study indicated that the contribution rate of *Gigaspora* OTU4 to plant growth in the native soil was the least among the soil pH gradient. Most previous work demonstrated that the AM fungal contribution to host growth under stress conditions is higher than that under normal conditions (Feng et al., 2020b), which supports the results in this study. Differently, *Sphingomonas* OTU3 significantly increased the shoot biomass in three acidic soils (pH = 3.44, 4.17, 4.85) but slightly decreased it in the neutral soil. *Sphingomonas* is a widely distributed bacterial taxon with multi-functions (Asaf et al., 2020). Most previous studies demonstrated that *Sphingomonas* isolates are an effective degrader of diverse organic pollutants (Li et al., 2017, 2020). However, a recent experiment revealed that *Sphingomonas* isolates promote plant growth independent of stress (Luo et al., 2019). We did not explore the mechanisms by which this isolate promoted plant growth in this study. Luo et al. (2019) reported that rhizobacteria *Sphingomonas* sp. Cra20 increased the biomass of *Arabidopsis thaliana* by improving the root architecture (e.g., more lateral roots and root hairs). Some *Sphingomonas* isolates are endophytes, promoting plant growth by producing phytohormones or fixing nitrogen (Videira et al., 2009; Khan et al., 2014).

We observed a negative interaction between the fungus and bacterium in this study. A suppression of AM fungi by soil microbes has been reported (Svenningsen et al., 2018). Intriguingly, a positive interaction of *Gigaspora* OTU4 and *Sphingomonas* OTU3 was observed only in the native soil, which might be the important reason why bahiagrass adapts to the native conditions. Zhang et al. (2016) described the cooperation of AM fungus and phosphate-solubilizing bacterium (PSB) in mobilizing organic phosphorus, where the glucose secreted by AM fungus acted as signaling molecules to promote the functioning of PSB. In this study, whether *Gigaspora* OTU4 and *Sphingomonas* OTU3 exerted a cooperative effect on plant growth is open to be explored in the future.

### Response of the microbial community to the pH gradient

Soil pH is one of the most important abiotic factors regulating microbial community. In this study, soil pH was set as the only variable and then was verified to be the sole edaphic factor shaping the AM fungal community and the top factor shaping the bacterial community. This result is consistent with the previous reports (Lauber et al., 2009; Shen et al., 2013). Hazard et al. (2013) indicated that the soil pH was the important factor driving the shift in the AM fungal community in Ireland pasture soils. In a global scale investigation, soil pH was identified as the key factor shaping the bacterial community, followed by the SOM and soil water content. Those factors other than soil pH affected the bacterial community but not the AM fungal community, probably indicating the higher sensitivity of bacteria to edaphic factors than AM fungi (Singh et al., 2008). Actually, AM fungi, as a symbiotic microbe, depend on the host plant community more than on the soil physicochemical properties (Krüger et al., 2017). However, only one plant species was used as a host in this study, and thus, the soil pH strongly shaped the AM fungal community.

### Seeking the beneficial microbial isolates with the aid of high-throughput sequencing

Utilizing beneficial microbes to promote crop production is increasingly recognized as a favorable strategy, especially in sustainable agricultural systems (Prasad et al., 2019). For this purpose, identifying and isolating the beneficial microbes are necessary but frustrating steps. In this study, we established for the first time a pipeline to effectively identify the growth-promoting isolates, which was guided by the high-throughput sequencing. In this pipeline, establishing a series of growth performance is the perquisite if the growth-promoting microbes are of interest. Then, a linear regression model between the growth performance and microbial OTU abundances based on high-throughput sequencing is necessary to identify the OTU, whose abundance is positively correlated with the growth performance. Those OTUs with high abundances were selected in this study because predominant microbes are more competitive to colonize the native soil than those with low abundances (Jiao et al., 2019).

Spores but not hyphae in soil are the most reliable propagules in isolating AM fungi from environments because the pure culture of AM fungi is not available yet (Liu et al., 2019). Moreover, the spore morphology is characteristic of AM fungal taxa, which is even superior to molecular methods (Wetzel et al., 2014). Similarly, R2A medium is regarded as optimal for isolating *Sphingomonas* (Vanbroekhoven et al., 2004). Therefore, isolating the identified beneficial isolates, e.g., *Gigaspora* OTU4 and *Sphingomonas* OTU3 in this study, was relatively easy. However, isolating other beneficial microbes can be challenging because more than 99% microbes in environments are presently unculturable and selective medium for a particular taxon is not always available (Pham and Kim, 2012). In this scenario, the development of efficient culture techniques and selective media is critical and urgent for the application of this pipeline.

### Dominant taxa or rare Taxa? It is a question

Since beneficial microbes can promote plant growth with diverse mechanisms, a tremendous amount of PGPRs have been isolated from various environments for decades (Bhattacharyya and Jha, 2012). However, a large proportion of the isolates are not effective in their native soils, partially due to the small size of their population after competition with other taxa (Strigul and Kravchenko, 2006). Manzoor et al. (2017) reported that some PSB isolates did not exhibit any effect in the native soil, although they were highly efficient in solubilizing phosphate on a plate. Collavino et al. (2010) compared two PSB isolates with high phosphate-solubilizing capacities in liquid culture and found that one isolate, *Burkholderia* sp. R4M-F, was ineffective in the native soil. This ineffectiveness can be attributed to the declined population size after application (Valetti et al., 2018). Soil conditions, e.g., pH, nutrient level, and SOM content, are the main determinants of the soil microbial community structure and functionality (Zhou et al., 2019). Along these lines, an isolate that is present in a given environment as a subordinate taxon cannot turn into a dominant taxon even if it is inoculated in a large quantity in the native soil after *in vitro* propagation. Therefore, it is reliable to isolate those isolates for a promising effect, which are present in the target environment as dominant taxa. In this study, we isolated the predominant taxa according to the high-throughput sequencing, which enabled the colonization of the target soil by both *Gigaspora* OTU3 and *Sphingomonas* OTU4 in large quantities.

## Conclusion

Using a high-throughput sequencing, we explored the AM fungal community and bacterial community associated with bahiagrass in soils with different pH levels (3.14∼8.59). The plant biomass ranked highest in the native soil with the original soil pH (pH 4.85), with both a decrease and an increase in the pH inhibiting plant growth. The abundances of some AM fungal and bacterial taxa were positively related with the plant biomass across the soil pH gradient. Accordingly, we identified the AM fungal and bacterial taxa, which were the most abundant and the most promotive to plant growth. The subsequent isolation revealed that *Gigaspora* OTU3 and *Sphingomonas* OTU4 were the targeted fungal and bacterial taxa. These two isolates efficiently promoted the plant growth and a positive interaction was observed between them. Consequently, this study developed a pipeline to screen for microbial isolates, which facilitated the growth of the host plants to the native soil with the original soil pH. In this pipeline, the high-throughput sequencing and regression analysis were essential to guide the isolation of targeted microbial taxa using plating, and the abundance of the targeted microbial taxa in the native soil was also considered. Moreover, the improvement of isolation techniques is critical for the isolation of targeted taxa because most microbes are presently unculturable.

## Data availability statement

The datasets presented in this study can be found in online repositories. The names of the repository/repositories and accession number(s) can be found in the article/Supplementary material.

## Author contributions

ZF, XL, YQ, GF, and YZ conducted the experiments and analyzed the data. ZF and XL wrote the manuscript. QY and HZ conceived the study and revised the manuscript. All authors read and approved the final manuscript.

## Footnotes

1
https://www2.dijon.inrae.fr/mychintec/Mycocalc-prg/download.html

2
https://invam.ku.edu/
