# Supplementary material for: Cooperation of arbuscular mycorrhizal fungi and bacteria to facilitate the host plant growth dependent on soil pH
Source: Front Microbiol. 2023 Feb 20;14:1116943. doi: 10.3389/fmicb.2023.1116943 (PMC9986299; doi:10.3389/fmicb.2023.1116943)
Supplement: Supplementary file 1 [file Image_1.PDF]

Supplementary Material

Cooperation of Arbuscular Mycorrhizal Fungi and Bacteria to Facilitate the Host Plant Growth Dependent on Soil pH

Zengwei Feng[1,2†], Xiaodi Liu[1†], Yongqiang Qin[2], Guangda Feng[1], Yang Zhou[1*], Honghui Zhu[1*] and Qing Yao[2*]

1 Key Laboratory of Agricultural Microbiomics and Precision Application (MARA), Guangdong Provincial Key Laboratory of Microbial Culture Collection and Application, Key Laboratory of Agricultural Microbiome (MARA), State Key Laboratory of Applied Microbiology Southern China, Institute of Microbiology, Guangdong Academy of Sciences, Guangzhou 510070, China

2 College of Horticulture, Guangdong Province Key Laboratory of Microbial Signals and Disease Control, Guangdong Engineering Research Center for Litchi, South China Agricultural University, Guangzhou 510642, China

*** Correspondence:**

Qing Yao

yaoqscau@scau.edu.cn

Honghui Zhu

zhuhh@gdim.cn

Yang Zhou

zhouyang@gdim.cn

[†]These authors contributed equally to this work.

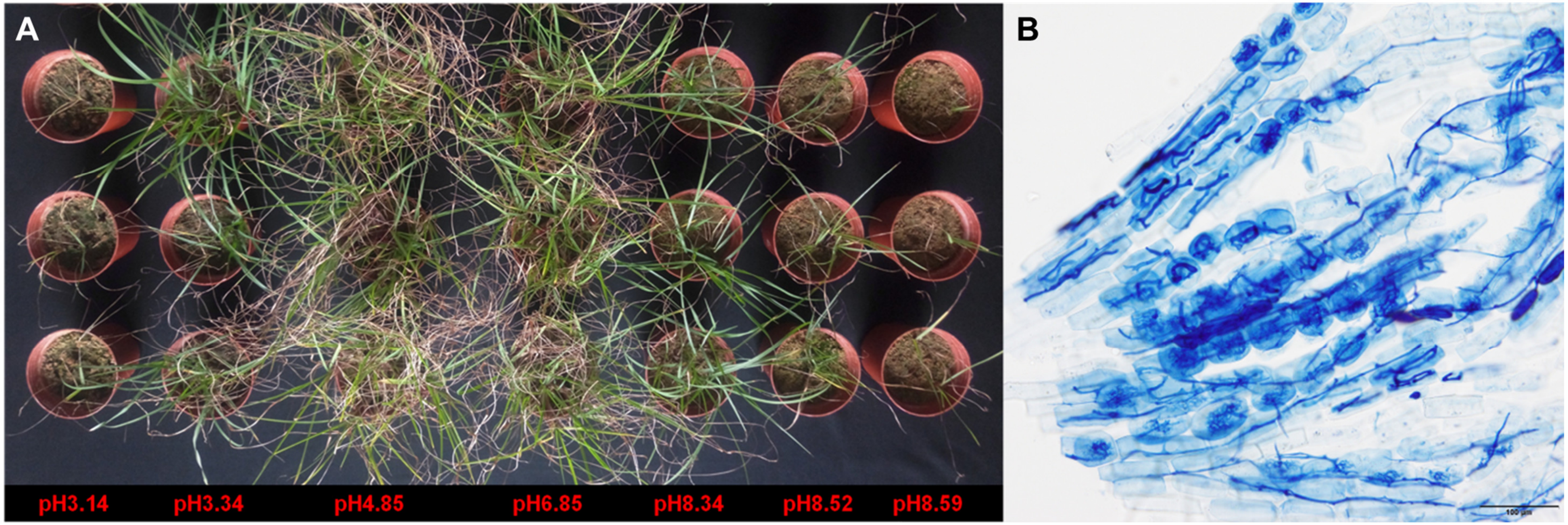

Supplementary Figure 1. The effects of the soil pH gradient on plant growth and mycorrhizal colonization in roots. (A) plant growth performance as affected by different soil pH levels; (B) mycorrhizal colonization of bahiagrass roots by indigenous AM fungal community. The soil with pH = 4.85 was the original soil, while lower and higher pH gradients were achieved by applying sulfur and calcium hydroxide, respectively.

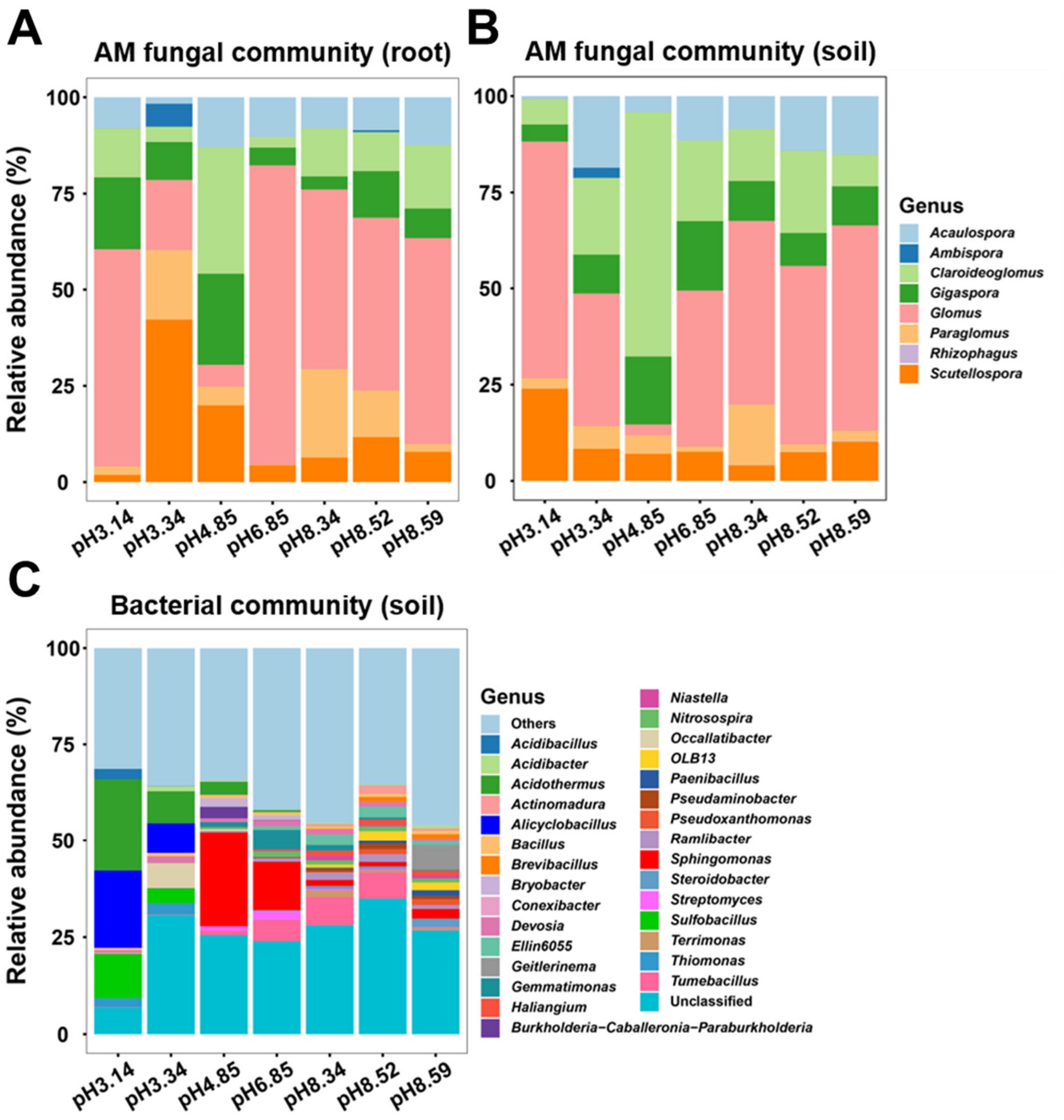

Supplementary Figure 2. Relative abundances of AM fungal and bacterial genera in soils and in roots as affected by different soil pH levels. (A) AM fungal community in roots; (B) AM fungal community in soils; (C) Bacterial community in soils. The soil with pH = 4.85 was the original soil, while lower and higher pH gradients were achieved by applying sulfur and calcium hydroxide, respectively.

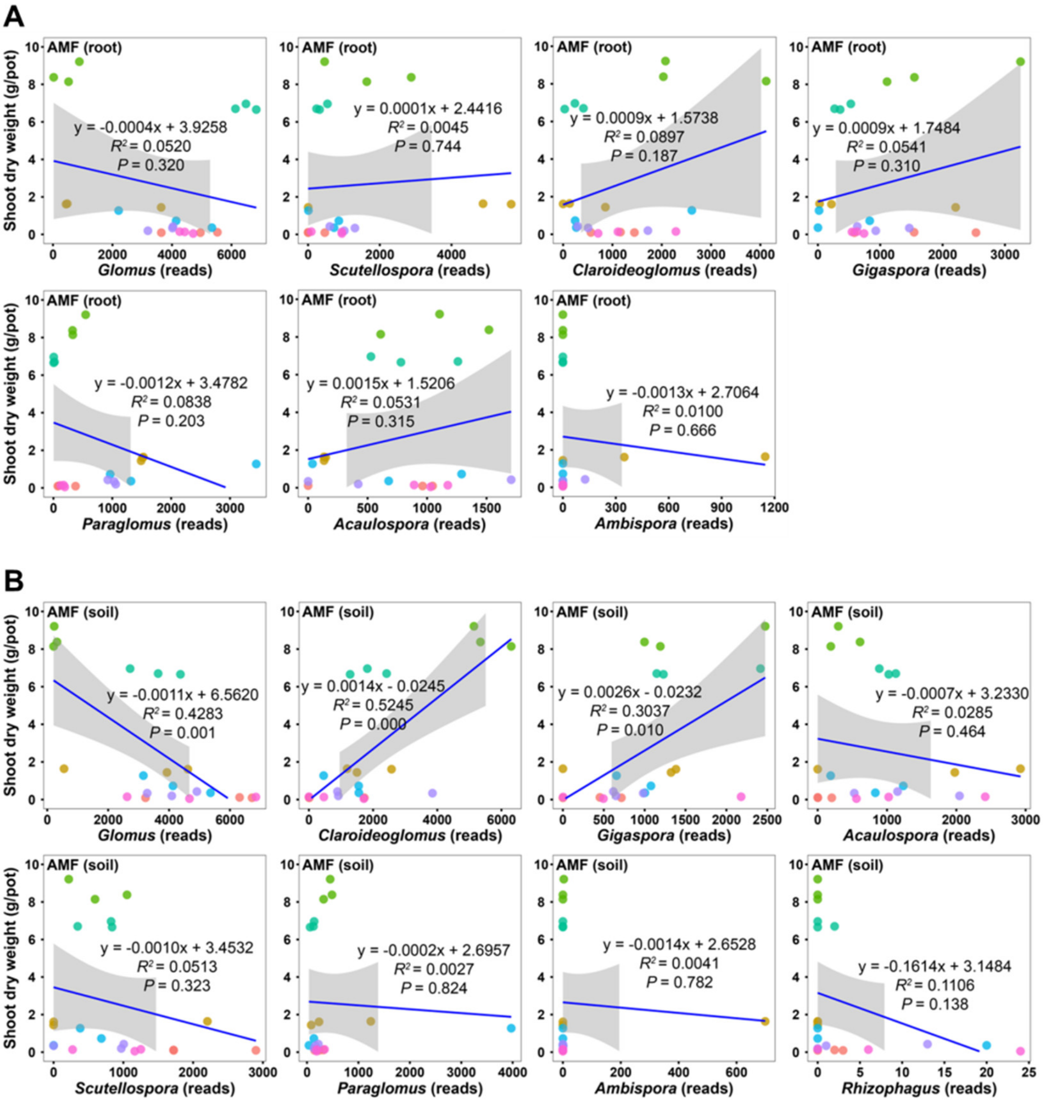

A
AMF (root)
y = -0.0004x + 3.9258
R² = 0.0520
P = 0.320
Glomus (reads)
AMF (root)
y = 0.0001x + 2.4416
R² = 0.0045
P = 0.744
Scutellospora (reads)
AMF (root)
y = 0.0009x + 1.5738
R² = 0.0897
P = 0.187
Claroideoglomus (reads)
AMF (root)
y = 0.0009x + 1.7484
R² = 0.0541
P = 0.310
Gigaspora (reads)
AMF (root)
y = -0.0012x + 3.4782
R² = 0.0838
P = 0.203
Paraglomus (reads)
AMF (root)
y = 0.0015x + 1.5206
R² = 0.0531
P = 0.315
Acaulospora (reads)
AMF (root)
y = -0.0013x + 2.7064
R² = 0.0100
P = 0.666
Ambispora (reads)
Shoot dry weight (g/pot)
B
AMF (soil)
y = -0.0011x + 6.5620
R² = 0.4283
P = 0.001
Glomus (reads)
AMF (soil)
y = 0.0014x - 0.0245
R² = 0.5245
P = 0.000
Claroideoglomus (reads)
AMF (soil)
y = 0.0026x - 0.0232
R² = 0.3037
P = 0.010
Gigaspora (reads)
AMF (soil)
y = -0.0007x + 3.2330
R² = 0.0285
P = 0.464
Acaulospora (reads)
AMF (soil)
y = -0.0010x + 3.4532
R² = 0.0513
P = 0.323
Scutellospora (reads)
AMF (soil)
y = -0.0002x + 2.6957
R² = 0.0027
P = 0.824
Paraglomus (reads)
AMF (soil)
y = -0.0014x + 2.6528
R² = 0.0041
P = 0.782
Ambispora (reads)
AMF (soil)
y = -0.1614x + 3.1484
R² = 0.1106
P = 0.138
Rhizophagus (reads)
Shoot dry weight (g/pot)

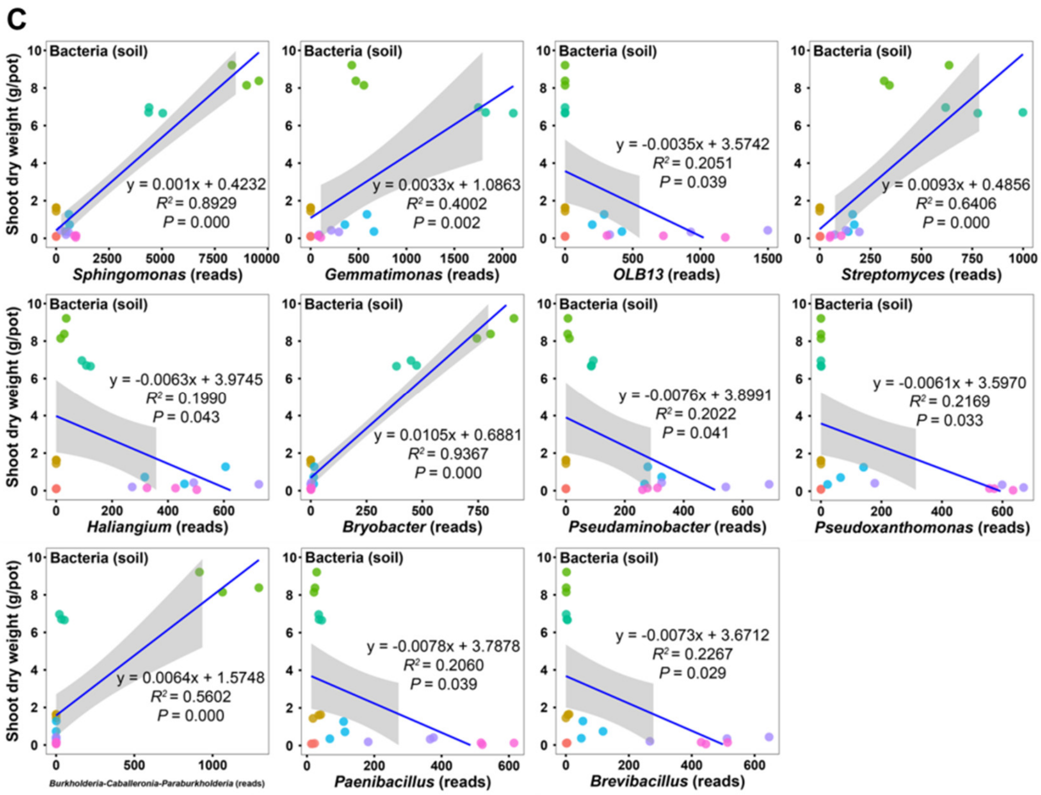

Supplementary Figure 3. Regression analysis between the shoot dry weight and abundances (reads number) of AM fungal and bacterial genera in soils and roots. For AM fungi, all genera were included, while for bacteria, only those bacterial genera with significant relationship (P < 0.05) were included. (A) shoot dry weight *vs.* abundance of AM fungal genera in roots; (B) shoot dry weight *vs.* abundance of AM fungal genera in soil; (C) shoot dry weight *vs.* abundance of bacterial genera in soil.

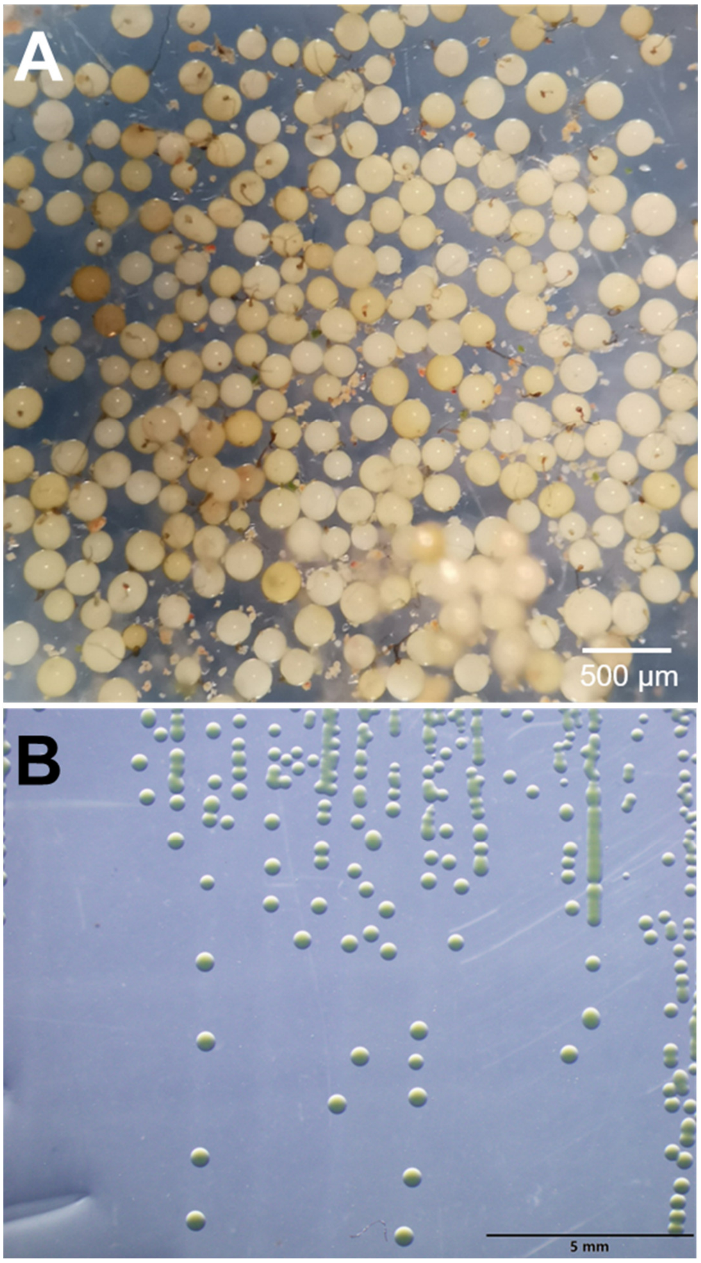

Supplementary Figure 4. The spores of *Gigaspora* OTU4 (A) and the colony of *Sphingomonas* OTU3 (B) on R2A medium.

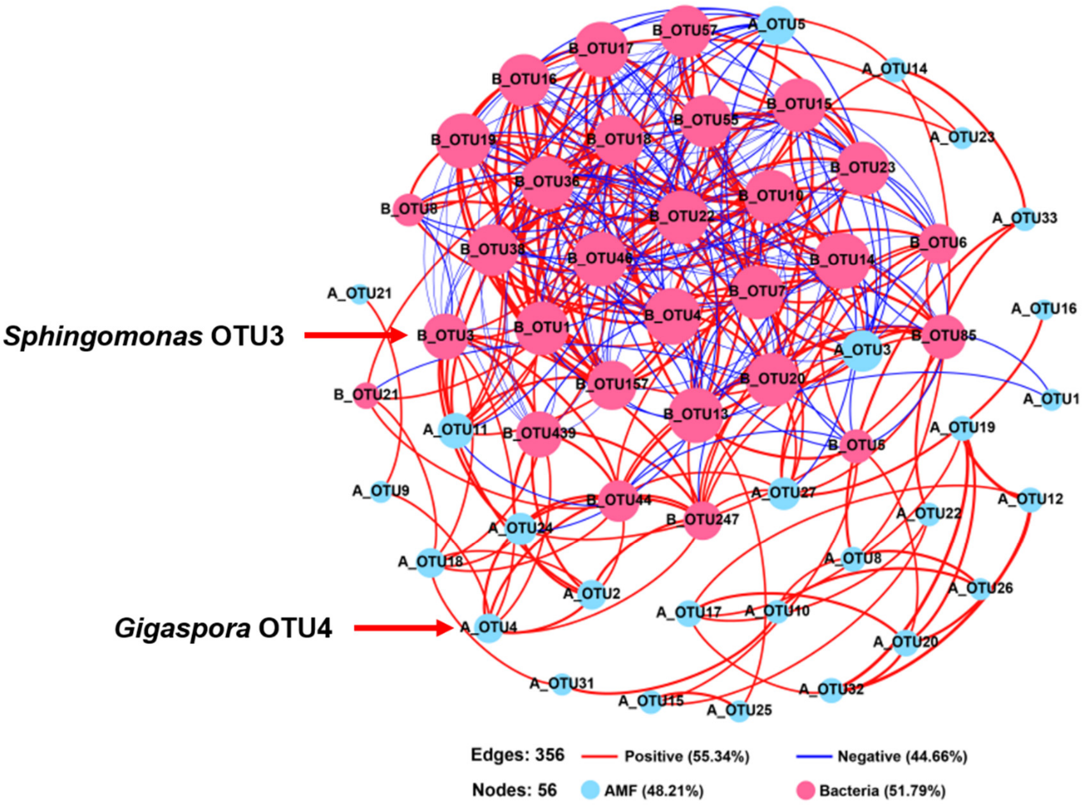

Supplementary Figure 5. Network analysis of the AM fungal OTUs and the bacterial OTUs in soils. *Gigaspora* OTU4 and *Sphingomonas* OTU3 are highlighted with red arrows.
