# Supplementary material for: Cooperation of arbuscular mycorrhizal fungi and bacteria to facilitate the host plant growth dependent on soil pH
Source: Front Microbiol. 2023 Feb 20;14:1116943. doi: 10.3389/fmicb.2023.1116943 (PMC9986299; doi:10.3389/fmicb.2023.1116943)
Supplement: Supplementary file 2 [file Table_1.DOCX]

***Supplementary Material***

**Cooperation of Arbuscular Mycorrhizal Fungi and Bacteria to Facilitate the Host Plant Growth Dependent on Soil pH**

**Zengwei Feng^1,2†^, Xiaodi Liu^1†^, Yongqiang** **Qin^2^, Guangda Feng^1^, Yang Zhou^1*^, Honghui Zhu^1*^ and Qing Yao^2*^**

1 Key Laboratory of Agricultural Microbiomics and Precision Application (MARA), Guangdong Provincial Key Laboratory of Microbial Culture Collection and Application, Key Laboratory of Agricultural Microbiome (MARA), State Key Laboratory of Applied Microbiology Southern China, Institute of Microbiology, Guangdong Academy of Sciences, Guangzhou 510070, China

2 College of Horticulture, Guangdong Province Key Laboratory of Microbial Signals and Disease Control, Guangdong Engineering Research Center for Litchi, South China Agricultural University, Guangzhou 510642, China

*** Correspondence:**

Qing Yao

yaoqscau@scau.edu.cn

Honghui Zhu

zhuhh@gdim.cn

Yang Zhou

zhouyang@gdim.cn

**^†^**These authors contributed equally to this work.

**Supplementary Table 1.** Canonical correspondence analysis (CCA) of the effects of the soil chemical properties on the microbial community (at OTU level). Initial pH, the soil pH before planting bahiagrass; Final pH, the soil pH after planting bahiagrass for four months; SOM, soil organic matter; TN, total nitrogen; TP, total phosphorus; TK, total potassium; AN, available nitrogen; AP, available phosphorus; AK, available potassium; ECa, exchangeable calcium; AS, available sulphur; EAl, exchangeable aluminum. The soil chemical properties which showed significant effect on microbial community are highlighted in red.

| Soil chemical properties | CCA1 | CCA2 | *R^2^* | *P* values | Significance |
| --- | --- | --- | --- | --- | --- |
|  | AM fungal community in roots | | | | |
| Initial pH | 0.9805 | -0.1964 | 0.4755 | 0.002 | ** |
| Final pH | 0.9735 | -0.2288 | 0.4715 | 0.002 | ** |
| EAl | -0.9924 | 0.1227 | 0.2242 | 0.103 |  |
| ECa | 0.9907 | -0.1363 | 0.1546 | 0.183 |  |
| AP | 0.5482 | -0.8364 | 0.1393 | 0.272 |  |
| AN | -0.9995 | 0.0307 | 0.1192 | 0.279 |  |
| AK | 0.9279 | -0.3728 | 0.0830 | 0.492 |  |
| AS | -0.9391 | 0.3436 | 0.0262 | 0.822 |  |
|  | AM fungal community in soils | | | | |
| Initial pH | -0.9508 | -0.3097 | 0.2598 | 0.035 | * |
| Final pH | -0.9437 | -0.3307 | 0.2549 | 0.040 | * |
| EAl | -0.9717 | -0.2363 | 0.1912 | 0.125 |  |
| ECa | -0.9991 | -0.0418 | 0.1315 | 0.265 |  |
| AP | 0.9988 | 0.0490 | 0.0971 | 0.363 |  |
| AN | -0.5804 | -0.8143 | 0.0227 | 0.832 |  |
| AK | -0.2648 | -0.9643 | 0.0107 | 0.921 |  |
| AS | 0.6069 | -0.7948 | 0.0049 | 0.980 |  |
|  | Bacterial community in soils | | | | |
| Initial pH | 0.9290 | 0.3702 | 0.9929 | 0.001 | *** |
| Final pH | 0.9231 | 0.3845 | 0.9914 | 0.001 | *** |
| EAl | -0.9997 | 0.0231 | 0.9455 | 0.001 | *** |
| ECa | -0.0474 | 0.9989 | 0.8253 | 0.001 | *** |
| AP | -0.9955 | 0.0949 | 0.8142 | 0.001 | *** |
| AN | 0.1753 | 0.9845 | 0.8060 | 0.001 | *** |
| AK | 0.7219 | 0.6920 | 0.7078 | 0.001 | *** |
| AS | -0.9560 | 0.2934 | 0.6987 | 0.001 | *** |

**Supplementary Table 2.** Alpha diversity of microbial community in roots and in soils across the soil pH gradient. Soil with the pH = 4.85 was the original soil. Values (mean ± standard error, *n* = 3) with different letters are significantly different (*P* < 0.05) by Tukey’s HSD test.

| Soil pH | OTUs | Chao1 index | Shannon-wiener | Simpson index |
| --- | --- | --- | --- | --- |
|  | AM fungi community in roots | | | |
| pH = 3.14 | 32.00 bc | 36.98 b | 1.76 ab | 0.74 a |
| pH = 3.34 | 31.33 bc | 38.00 b | 1.58 ab | 0.67 ab |
| pH = 4.85 | 59.33 a | 86.04 a | 2.21 a | 0.81 a |
| pH = 6.85 | 48.00 ab | 61.28 ab | 1.26 b | 0.48 b |
| pH = 8.34 | 31.00 c | 40.78 b | 1.65 ab | 0.69 a |
| pH = 8.52 | 35.33 bc | 42.33 b | 2.10 a | 0.81 a |
| pH = 8.59 | 33.67 bc | 39.65 b | 1.75 ab | 0.71 a |
|  | AM fungi community in soils | | | |
| pH = 3.14 | 27.00 b | 33.67 a | 1.27 b | 0.59 b |
| pH = 3.34 | 33.00 b | 46.50 a | 1.84 ab | 0.77 ab |
| pH = 4.85 | 61.33 a | 74.28 a | 1.66 ab | 0.61 ab |
| pH = 6.85 | 64.00 a | 69.74 a | 2.37 a | 0.85 a |
| pH = 8.34 | 35.00 b | 42.86 a | 1.85 ab | 0.74 ab |
| pH = 8.52 | 37.00 b | 47.64 a | 2.03 ab | 0.80 ab |
| pH = 8.59 | 37.33 b | 55.83 a | 1.81 ab | 0.74 ab |
|  | Bacterial community in soils | | | |
| pH = 3.14 | 147.67 d | 194.00 d | 3.14 b | 0.92 b |
| pH = 3.34 | 181.33 d | 215.33 d | 3.26 b | 0.92 b |
| pH = 4.85 | 559.33 c | 647.00 c | 4.73 a | 0.96 ab |
| pH = 6.85 | 752.67 a | 861.33 a | 5.27 a | 0.99 a |
| pH = 8.34 | 720.33 a | 832.00 a | 4.98 a | 0.98 a |
| pH = 8.52 | 653.00 b | 741.67 b | 4.72 a | 0.96 ab |
| pH = 8.59 | 561.33 c | 628.67 c | 4.82 a | 0.97 a |

**Supplementary Table 3.** Regression equations between OTU abundance and shoot biomass, and relative abundances of the top 30 AM fungal OTUs in roots. The OTUs with significant and positive relation with shoot biomass are highlighted in red.

| AM fungal OTUs | Closest taxa | Average abundance | Regression equation | *P* value | *R^2^* | pH  3.14 | pH  3.34 | pH  4.85 | pH  6.85 | pH  8.34 | pH  8.52 | pH  8.59 |
| --- | --- | --- | --- | --- | --- | --- | --- | --- | --- | --- | --- | --- |
| OTU1 | Glomus_VTX00113 | 3066 | y=-0.0003x+3.5635 | 0.432 | 0.033 | 37.53 | 17.34 | 2.36 | 70.84 | 43.67 | 37.58 | 48.39 |
| OTU2 | Claroideoglomus_VTX00278 | 754 | y=0.0013x+1.6045 | 0.061 | 0.173 | 11.62 | 0.00 | 31.88 | 1.29 | 1.10 | 5.04 | 12.41 |
| OTU4 | Gigaspora_VTX00039 | 586 | y=0.0014x+1.8036 | 0.118 | 0.124 | 14.95 | 8.60 | 19.16 | 3.88 | 1.57 | 0.00 | 1.13 |
| OTU45 | Glomus_VTX00130 | 494 | y=-0.0001x+2.6733 | 0.806 | 0.003 | 0.01 | 37.20 | 3.10 | 0.00 | 0.00 | 0.00 | 1.18 |
| OTU5 | Acaulospora_VTX00026 | 477 | y=-0.0011x+3.1284 | 0.499 | 0.024 | 7.92 | 1.62 | 1.59 | 9.01 | 5.52 | 6.66 | 7.79 |
| OTU6 | Paraglomus_VTX00281 | 222 | y=-0.0006x+2.7498 | 0.543 | 0.020 | 0.08 | 0.80 | 0.01 | 0.10 | 16.79 | 0.72 | 0.18 |
| OTU23 | Scutellospora_VTX00041 | 217 | y=0.0035x+1.8499 | 0.075 | 0.157 | 0.00 | 0.00 | 9.34 | 1.16 | 0.00 | 6.07 | 1.69 |
| OTU47 | Glomus_VTX00310 | 196 | y=0.0010x+2.4120 | 0.671, | 0.010 | 1.76 | 6.03 | 4.68 | 0.01 | 0.05 | 2.82 | 1.17 |
| OTU8 | Glomus_VTX00310 | 156 | y=-0.0002x+2.6384 | 0.891 | 0.001 | 9.64 | 0.36 | 2.93 | 0.18 | 0.01 | 0.00 | 0.00 |
| OTU3 | Paraglomus_VTX00238 | 137 | y=-0.0010x+2.7400 | 0.610 | 0.014 | 0.01 | 10.88 | 0.01 | 0.00 | 0.62 | 0.01 | 0.01 |
| OTU35 | Claroideoglomus_VTX00278 | 127 | y=-0.0044x+3.1627 | 0.132 | 0.115 | 0.01 | 0.00 | 0.00 | 0.00 | 3.96 | 6.74 | 0.00 |
| OTU29 | Acaulospora_VTX00028 | 121 | y=0.0079x+1.6466 | 0.002 | 0.400 | 0.00 | 0.03 | 7.80 | 0.48 | 1.90 | 0.00 | 0.00 |
| OTU27 | Claroideoglomus_VTX00193 | 116 | y=-0.0006x+2.6715 | 0.699 | 0.008 | 0.00 | 0.00 | 0.00 | 0.03 | 9.69 | 0.00 | 0.00 |
| OTU7 | Scutellospora_VTX00041 | 116 | y=0.0003x+2.5750 | 0.949 | 0.000 | 1.79 | 0.04 | 0.66 | 2.93 | 0.04 | 0.00 | 4.26 |
| OTU9 | Claroideoglomus_VTX00193 | 57 | y=-0.0023x+2.7400 | 0.717 | 0.007 | 0.96 | 0.15 | 0.01 | 1.26 | 0.04 | 1.97 | 0.42 |
| OTU10 | Gigaspora_VTX00039 | 56 | y=-0.0020x+2.7188 | 0.506 | 0.024 | 0.00 | 0.00 | 0.01 | 0.00 | 0.00 | 4.70 | 0.00 |
| OTU15 | Glomus_VTX00310 | 55 | y=-0.0044x+2.8500 | 0.316 | 0.053 | 2.58 | 0.00 | 0.00 | 0.02 | 0.00 | 2.06 | 0.00 |
| OTU49 | Claroideoglomus_VTX00278 | 52 | y=-0.0019x+2.7062 | 0.668 | 0.010 | 0.00 | 4.05 | 0.00 | 0.00 | 0.00 | 0.32 | 0.00 |
| OTU16 | Acaulospora_VTX00026 | 51 | y=-0.0024x+2.7310 | 0.497 | 0.025 | 0.00 | 0.00 | 0.01 | 0.21 | 0.04 | 0.06 | 4.00 |
| OTU41 | Paraglomus_VTX00238 | 50 | y=-0.0041x+2.8104 | 0.402 | 0.037 | 0.00 | 0.00 | 0.03 | 0.00 | 4.15 | 0.00 | 0.00 |
| OTU28 | Gigaspora_VTX00039 | 46 | y=-0.0062x+2.8888 | 0.353 | 0.046 | 0.00 | 0.00 | 0.03 | 0.23 | 1.83 | 0.00 | 1.76 |
| OTU13 | Claroideoglomus_VTX00278 | 45 | y=-0.0032x+2.7467 | 0.460 | 0.029 | 0.00 | 0.37 | 0.04 | 0.00 | 0.00 | 0.00 | 3.33 |
| OTU18 | Gigaspora_VTX00039 | 44 | y=0.0175x+1.8306 | 0.012 | 0.291 | 0.00 | 0.24 | 2.57 | 0.19 | 0.00 | 0.71 | 0.00 |
| OTU24 | Glomus_VTX00310 | 44 | y=-0.0048x+2.8160 | 0.433 | 0.033 | 3.34 | 0.00 | 0.30 | 0.05 | 0.00 | 0.00 | 0.00 |
| OTU65 | Claroideoglomus_VTX00193 | 43 | y=-0.0028x+2.7258 | 0.479 | 0.027 | 0.00 | 0.00 | 0.00 | 0.00 | 0.00 | 3.61 | 0.00 |
| OTU38 | Glomus_VTX00222 | 42 | y=-0.0056x+2.8377 | 0.443 | 0.031 | 0.00 | 2.05 | 0.01 | 0.00 | 0.00 | 1.43 | 0.00 |
| OTU62 | Claroideoglomus_VTX00278 | 40 | y=-0.0014x+2.6622 | 0.741 | 0.006 | 0.00 | 3.37 | 0.02 | 0.00 | 0.00 | 0.00 | 0.00 |
| OTU68 | Acaulospora_VTX00028 | 38 | y=-0.0018x+2.6751 | 0.752 | 0.005 | 0.00 | 0.00 | 0.60 | 0.01 | 0.00 | 0.12 | 2.46 |
| OTU48 | Ambispora_VTX00242 | 32 | y=-0.0038x+2.7282 | 0.473 | 0.027 | 0.00 | 0.00 | 0.02 | 0.00 | 0.00 | 0.00 | 2.68 |
| OTU20 | Glomus_VTX00067 | 29 | y=0.0071x+2.4005 | 0.222 | 0.078 | 0.00 | 0.00 | 0.00 | 2.44 | 0.00 | 0.00 | 0.00 |

**Supplementary Table 4.** Regression equations between OTU abundance and shoot biomass, and relative abundances of the top 30 AM fungal OTUs in soils. The OTUs with significant and positive relation with shoot biomass are highlighted in red.

| AM fungal OTUs | Closest taxa | Average abundance | Regression equation | *P* value | *R^2^* | pH  3.14 | pH  3.34 | pH  4.85 | pH  6.85 | pH  8.34 | pH  8.52 | pH  8.59 |
| --- | --- | --- | --- | --- | --- | --- | --- | --- | --- | --- | --- | --- |
| OTU1 | Glomus_VTX00113 | 2690 | y=-0.0011x+5.4579 | 0.005 | 0.343 | 56.57 | 30.60 | 2.18 | 25.16 | 36.96 | 31.04 | 31.22 |
| OTU2 | Claroideoglomus_VTX00278 | 1258 | y=0.0012x+1.0989 | 0.001 | 0.440 | 6.21 | 0.00 | 59.51 | 8.69 | 5.08 | 14.11 | 6.33 |
| OTU4 | Gigaspora_VTX00039 | 646 | y=0.0037x+0.2123 | 0.000 | 0.486 | 3.75 | 5.07 | 14.86 | 15.20 | 2.56 | 8.08 | 1.81 |
| OTU5 | Acaulospora_VTX00026 | 559 | y=-0.0005x+2.9028 | 0.617 | 0.013 | 0.73 | 7.51 | 0.74 | 10.47 | 4.42 | 9.35 | 11.16 |
| OTU7 | Scutellospora_VTX00041 | 214 | y=0.0001x+2.5854 | 0.969 | 0.000 | 1.76 | 3.87 | 0.34 | 5.13 | 0.00 | 3.60 | 2.30 |
| OTU9 | Claroideoglomus_VTX00193 | 214 | y=0.0016x+2.2676 | 0.306 | 0.055 | 0.01 | 6.34 | 0.00 | 9.28 | 1.36 | 0.00 | 0.00 |
| OTU6 | Paraglomus_VTX00281 | 200 | y=-0.0003x+2.6734 | 0.709 | 0.008 | 0.10 | 0.14 | 0.01 | 0.45 | 14.96 | 0.19 | 0.07 |
| OTU10 | Gigaspora_VTX00039 | 194 | y=-0.0015x+2.8911 | 0.336 | 0.049 | 0.00 | 4.63 | 0.05 | 0.04 | 2.77 | 0.28 | 7.67 |
| OTU11 | Scutellospora_VTX00041 | 178 | y=-0.0012x+2.8170 | 0.334 | 0.049 | 14.17 | 0.00 | 0.00 | 0.00 | 0.00 | 0.00 | 0.00 |
| OTU3 | Paraglomus_VTX00238 | 167 | y=0.0027x+2.1607 | 0.348 | 0.046 | 0.96 | 5.57 | 4.65 | 0.00 | 0.00 | 0.47 | 1.63 |
| OTU8 | Glomus_VTX00310 | 136 | y=-0.0017x+2.8391 | 0.339 | 0.048 | 0.02 | 0.00 | 0.10 | 0.07 | 0.00 | 4.17 | 6.42 |
| OTU13 | Claroideoglomus_VTX00278 | 129 | y=-0.0015x+2.8047 | 0.475 | 0.027 | 0.00 | 5.44 | 0.04 | 0.01 | 1.60 | 3.20 | 0.00 |
| OTU16 | Acaulospora_VTX00026 | 122 | y=-0.0004x+2.6539 | 0.783 | 0.004 | 0.00 | 9.37 | 0.02 | 0.12 | 0.00 | 0.15 | 0.00 |
| OTU15 | Glomus_VTX00310 | 115 | y=-0.0011x+2.7313 | 0.466 | 0.028 | 0.00 | 0.01 | 0.10 | 0.05 | 0.00 | 0.00 | 9.02 |
| OTU14 | Acaulospora_VTX00026 | 106 | y=-0.0024x+2.8653 | 0.325 | 0.051 | 0.01 | 0.01 | 0.07 | 0.16 | 0.17 | 4.32 | 3.71 |
| OTU20 | Glomus_VTX00067 | 83 | y=0.0025x+2.3994 | 0.221 | 0.078 | 0.01 | 0.00 | 0.00 | 6.61 | 0.01 | 0.00 | 0.00 |
| OTU17 | Glomus_VTX00410 | 81 | y=-0.0029x+2.8376 | 0.318 | 0.052 | 4.62 | 0.01 | 0.00 | 0.00 | 0.04 | 1.78 | 0.00 |
| OTU22 | Scutellospora_VTX00255 | 79 | y=-0.0016x+2.7293 | 0.468 | 0.028 | 6.24 | 0.00 | 0.02 | 0.02 | 0.00 | 0.00 | 0.00 |
| OTU21 | Scutellospora_VTX00041 | 68 | y=-0.0034x+2.8337 | 0.347 | 0.047 | 0.97 | 0.00 | 0.03 | 0.04 | 0.00 | 3.65 | 0.71 |
| OTU23 | Scutellospora_VTX00041 | 62 | y=0.0197x+1.3750 | 0.002 | 0.396 | 0.00 | 0.00 | 2.05 | 1.61 | 1.29 | 0.00 | 0.00 |
| OTU18 | Gigaspora_VTX00039 | 60 | y=0.0046x+2.3334 | 0.384 | 0.040 | 0.00 | 0.00 | 1.14 | 1.16 | 2.43 | 0.00 | 0.00 |
| OTU24 | Glomus_VTX00310 | 58 | y=-0.0033x+2.7947 | 0.381 | 0.041 | 0.00 | 0.00 | 0.00 | 0.00 | 3.48 | 0.00 | 1.12 |
| OTU25 | Scutellospora_VTX00255 | 57 | y=-0.0021x+2.7270 | 0.477 | 0.027 | 0.00 | 0.00 | 0.04 | 0.00 | 0.00 | 0.00 | 4.46 |
| OTU26 | Claroideoglomus_VTX00278 | 55 | y=0.0030x+2.4398 | 0.499 | 0.024 | 0.00 | 2.87 | 1.52 | 0.01 | 0.00 | 0.00 | 0.00 |
| OTU32 | Acaulospora_VTX00026 | 45 | y=-0.0021x+2.7002 | 0.579 | 0.017 | 0.00 | 0.00 | 0.00 | 0.00 | 3.59 | 0.00 | 0.01 |
| OTU27 | Claroideoglomus_VTX00193 | 45 | y=0.0002x+2.5948 | 0.974 | 0.000 | 0.00 | 0.00 | 0.00 | 1.37 | 0.00 | 0.50 | 1.71 |
| OTU33 | Glomus_VTX00126 | 45 | y=-0.0022x+2.7031 | 0.585 | 0.016 | 0.00 | 0.00 | 0.17 | 0.00 | 0.00 | 3.39 | 0.00 |
| OTU31 | Glomus_VTX00099 | 42 | y=-0.0016x+2.6727 | 0.695 | 0.008 | 0.00 | 0.01 | 0.00 | 0.00 | 3.34 | 0.00 | 0.00 |
| OTU30 | Glomus_VTX00222 | 42 | y=-0.0014x+2.6634 | 0.734 | 0.006 | 0.00 | 3.35 | 0.00 | 0.00 | 0.00 | 0.00 | 0.00 |
| OTU34 | Glomus_VTX00125 | 42 | y=-0.0024x+2.7045 | 0.617 | 0.013 | 0.00 | 0.00 | 0.00 | 0.49 | 0.00 | 2.84 | 0.00 |

**Supplementary Table 5.** Regression equations between OTU abundance and shoot biomass, and relative abundances of the top 30 bacterial OTUs in soils. The OTUs with significant and positive relation with shoot biomass are highlighted in red.

| Bacterial OTUs | Closest taxa | Average abundance | Regression equation | *P* value | R^2^ | pH  3.14 | pH  3.34 | pH  4.85 | pH  6.85 | pH  8.34 | pH  8.52 | pH  8.59 |
| --- | --- | --- | --- | --- | --- | --- | --- | --- | --- | --- | --- | --- |
| OTU1 | o_Clostridiales | 1637 | y=-0.0004x+3.2623 | 0.129 | 0.117 | 18.60 | 12.43 | 0.00 | 0.00 | 0.00 | 0.00 | 0.00 |
| OTU3 | g_Sphingomonas | 1101 | y=0.0015x+0.9463 | 0.000 | 0.815 | 0.00 | 0.00 | 15.29 | 5.04 | 0.22 | 0.07 | 0.26 |
| OTU4 | g_Acidothermus | 1025 | y=-0.0005x+3.0859 | 0.152 | 0.105 | 17.53 | 1.89 | 0.01 | 0.01 | 0.00 | 0.00 | 0.00 |
| OTU2 | c_Oxyphotobacteria | 831 | y=-0.0001x+2.6666 | 0.826 | 0.003 | 0.00 | 0.01 | 3.43 | 0.29 | 0.92 | 9.77 | 1.33 |
| OTU14 | f_Microscillaceae | 772 | y=-0.0011x+3.4588 | 0.075 | 0.157 | 0.00 | 0.00 | 0.00 | 0.37 | 6.87 | 0.79 | 6.62 |
| OTU7 | f_Rhodanobacteraceae | 769 | y=-0.0002x+2.7603 | 0.597 | 0.015 | 0.00 | 14.53 | 0.05 | 0.00 | 0.00 | 0.00 | 0.00 |
| OTU22 | g_Tumebacillus | 730 | y=-0.0010x+3.3463 | 0.098 | 0.137 | 0.00 | 0.00 | 0.00 | 0.00 | 7.21 | 6.60 | 0.01 |
| OTU8 | f_Acidobacteriaceae | 564 | y=-0.0003x+2.7535 | 0.606 | 0.014 | 0.00 | 10.69 | 0.00 | 0.00 | 0.00 | 0.00 | 0.00 |
| OTU10 | s_Actinobacterium BGR88 | 536 | y=-0.0011x+3.2103 | 0.210 | 0.081 | 5.64 | 3.95 | 0.56 | 0.01 | 0.00 | 0.00 | 0.00 |
| OTU157 | g_Sulfobacillus | 426 | y=-0.0016x+3.2776 | 0.121 | 0.122 | 5.06 | 3.01 | 0.00 | 0.00 | 0.00 | 0.00 | 0.00 |
| OTU15 | f_Sphingomonadaceae | 394 | y=-0.0025x+3.5702 | 0.130 | 0.116 | 0.00 | 0.00 | 0.02 | 0.93 | 2.75 | 2.74 | 1.02 |
| OTU6 | g_Occallatibacter | 387 | y=-0.0001x+2.6360 | 0.875 | 0.001 | 0.23 | 6.52 | 0.57 | 0.01 | 0.00 | 0.00 | 0.00 |
| OTU55 | f_Longimicrobiaceae | 340 | y=-0.0018x+3.2108 | 0.140 | 0.111 | 0.00 | 0.00 | 0.00 | 0.05 | 4.41 | 1.98 | 0.01 |
| OTU5 | g_Geitlerinema | 336 | y=-0.0005x+2.7879 | 0.367 | 0.043 | 0.00 | 0.00 | 0.00 | 0.00 | 0.00 | 0.00 | 6.37 |
| OTU18 | g_Alicyclobacillus | 317 | y=-0.0015x+3.0886 | 0.153 | 0.105 | 5.30 | 0.71 | 0.00 | 0.00 | 0.00 | 0.00 | 0.00 |
| OTU20 | o_Saccharimonadales | 314 | y=-0.0003x+2.6970 | 0.742 | 0.006 | 0.01 | 5.62 | 0.32 | 0.00 | 0.00 | 0.00 | 0.00 |
| OTU9 | g_Watanabea | 296 | y=-0.0020x+3.1912 | 0.131 | 0.116 | 3.87 | 0.95 | 0.02 | 0.04 | 0.34 | 0.32 | 0.07 |
| OTU465 | g_Sphingomonas | 291 | y=0.0052x+1.0871 | 0.000 | 0.717 | 0.00 | 0.00 | 4.21 | 0.95 | 0.07 | 0.03 | 0.26 |
| OTU32 | o_Chloroplast | 286 | y=-0.0034x+3.5892 | 0.039 | 0.206 | 0.00 | 0.00 | 0.01 | 0.01 | 1.25 | 1.74 | 2.41 |
| OTU13 | c_Anaerolineae | 281 | y=-0.0035x+3.5742 | 0.039 | 0.205 | 0.00 | 0.00 | 0.00 | 0.00 | 0.82 | 2.49 | 2.01 |
| OTU57 | g_Nitrosospira | 279 | y=0.0012x+2.2606 | 0.715 | 0.007 | 0.00 | 0.00 | 0.48 | 1.41 | 1.07 | 1.30 | 1.01 |
| OTU21 | g_Tumebacillus | 275 | y=0.0025x+1.9078 | 0.017 | 0.265 | 0.01 | 0.01 | 0.01 | 5.15 | 0.04 | 0.01 | 0.00 |
| OTU19 | g_Thiomonas | 272 | y=-0.0023x+3.2211 | 0.171 | 0.096 | 2.26 | 2.90 | 0.00 | 0.00 | 0.00 | 0.00 | 0.00 |
| OTU12 | g_Watanabea | 267 | y=-0.0044x+3.7763 | 0.034 | 0.215 | 0.00 | 0.00 | 0.00 | 0.15 | 1.27 | 2.50 | 1.14 |
| OTU17 | o_Clostridiales | 260 | y=-0.0025x+3.2611 | 0.139 | 0.111 | 2.67 | 2.25 | 0.00 | 0.00 | 0.00 | 0.00 | 0.00 |
| OTU44 | f_Xanthobacteraceae | 256 | y=0.0096x+0.1360 | 0.000 | 0.939 | 0.00 | 0.40 | 2.60 | 1.39 | 0.22 | 0.15 | 0.10 |
| OTU11 | o_Nostocales | 246 | y=0.0049x+1.3895 | 0.000 | 0.520 | 0.00 | 0.00 | 1.16 | 3.51 | 0.00 | 0.00 | 0.00 |
| OTU36 | s_Alicyclobacillus sp. | 229 | y=-0.0030x+3.2868 | 0.119 | 0.123 | 2.73 | 1.60 | 0.00 | 0.00 | 0.00 | 0.00 | 0.00 |
| OTU85 | o_Saccharimonadales | 227 | y=-0.0003x+2.6736 | 0.835 | 0.002 | 0.00 | 3.90 | 0.40 | 0.01 | 0.00 | 0.00 | 0.00 |
| OTU16 | g_Alicyclobacillus | 218 | y=-0.0014x+2.9006 | 0.267 | 0.064 | 3.72 | 0.40 | 0.00 | 0.00 | 0.00 | 0.00 | 0.00 |
